# Supplementary material for: Uranium transport in acidic brines under reducing conditions
Source: Nat Commun. 2018 Apr 16;9:1469. doi: 10.1038/s41467-018-03564-7 (PMC5902481; doi:10.1038/s41467-018-03564-7)
Supplement: Supplementary file 1 — Supplementary Information [file 41467_2018_3564_MOESM1_ESM.pdf]

Supplementary Materials for

Uranium transport in acidic brines under reducing conditions

Timofeev et al.

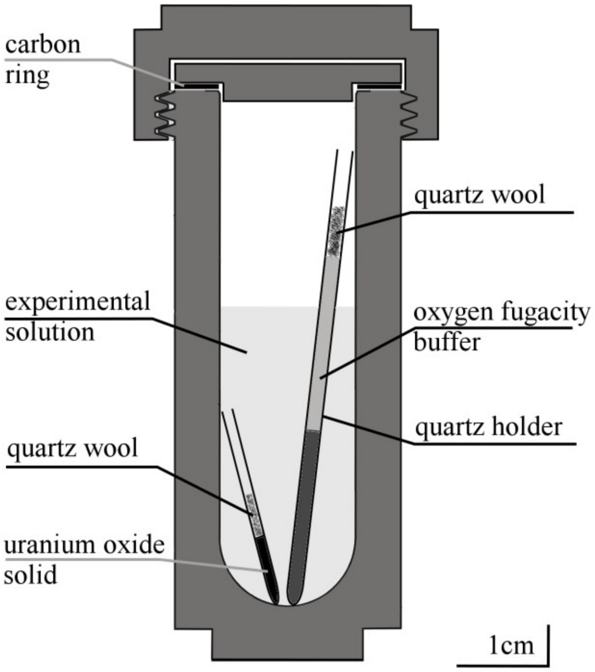

Supplementary Figure 1: A schematic diagram illustrating the experimental setup for $U_3O_8^{cryst}$ experiments and UO_2^{cryst} experiments at 300 and 350 °C. Malleable carbon rings provided an airtight seal upon closure of the autoclave. For experiments involving the Ni-NiO buffer, three new strands of nickel wire were placed into the extended quartz holder prior to the start of each experiment. Quartz wool was not added above nickel wires.

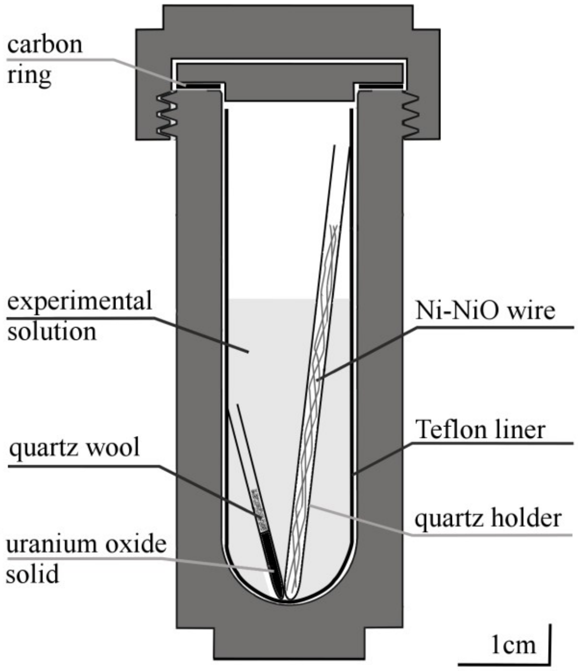

Supplementary Figure 2: A schematic diagram illustrating the experimental setup for UO_2^{cryst} experiments at 250 °C. The Ni-NiO wires described in the caption of Supplementary Figure 1 are shown in this diagram.

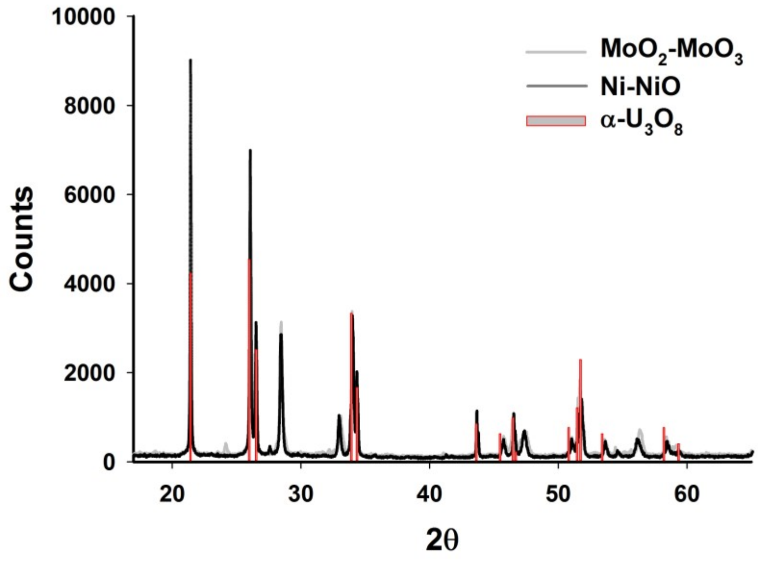

Supplementary Figure 3: X-ray diffraction (XRD) analyses of uranium oxide solids following completion of the experiments. Uranium oxide powders were chosen for analysis by XRD from initially $U_3O_8^{cryst}$ bearing oxidizing (MoO_2-MoO_3) and reducing (Ni-NiO) experiments. The close match between predicted XRD diffraction peaks for α-$U_3O_8^{cryst}$ and those observed in the experimental powders demonstrates that the powder remained largely unchanged.

Supplementary Table 1. Compositions of the experimental solutions. Concentrations are listed for solutions at 25 °C. The chloride activity for the temperature of interest is also listed. The data are organized in sections according to the oxygen fugacity buffer and uranium oxide solid employed in the experiments.

MoO_2-MoO_3, $U_3O_8^{cryst}$

T(°C)	NaCl(m)	HCl(m), 10^{-3}	aCl^- (T)	U(m), 10^{-7}	pH (25 °C)
300	1.00	13.7	0.20	1000	2.08
	1.00	13.4	0.18	822	2.09
	0.85	10.5	0.18	1030	2.19
	0.85	10.5	0.14	706	2.19
	0.55	8	0.13	236	2.29
	0.50	5.5	0.12	130	2.45
	0.40	8.1	0.085	172	2.27
	0.25	6.3	0.085	118	2.36
	0.25	6.3	0.074	61.5	2.36
	0.20	6.7	0.046	107	2.32
300	0.10	3.8	0.046	38.5	2.54
350	1.00	7.3	0.089	1040	2.35
	0.85	12.4	0.082	1510	2.12
	0.70	8.2	0.074	1510	2.29
	0.55	7.4	0.065	1020	2.32
	0.40	6.0	0.054	672	2.40
	0.40	5.7	0.054	352	2.42
	0.40	6.9	0.054	668	2.34
	0.25	6.9	0.042	503	2.32
	0.20	5.1	0.037	270	2.44
	0.060	3.4	0.018	153	2.57
	0.030	2.7	0.012	60.6	2.64
350	0.030	2.5	0.012	73.5	2.68

Ni-NiO, $U_3O_8^{cryst}$

T(°C)	NaCl(m)	HCl(m), 10^{-3}	aCl^- (T)	U(m), 10^{-7}	pH (25 °C)
250	1.00	10.6	0.31	808	2.19
	0.70	8.4	0.24	111	2.28
	0.55	8.9	0.20	219	2.24
	0.55	8.9	0.20	111	2.24
	0.40	6.6	0.16	17.0	2.36
	0.25	6.7	0.12	3.76	2.33
250	0.25	7.9	0.12	7.90	2.26
300	1.00	12.8	0.20	121	2.11
	1.00	11.7	0.20	147	2.15
	1.00	11.7	0.20	165	2.30
	0.85	10.0	0.18	37.5	2.41
	0.85	10.0	0.18	133	2.21
	0.70	8.6	0.16	25.5	2.27
	0.55	7.4	0.14	18.6	2.32
	0.55	7.4	0.14	22.3	2.45
	0.40	5.9	0.12	7.51	2.41
	0.40	7.5	0.12	13.2	2.30
	0.25	6.0	0.085	5.20	2.38
300	0.25	6.0	0.085	6.91	2.43
350	1.00	11.1	0.089	29.8	2.17
	1.00	11.6	0.089	62.5	2.15
	1.00	11.1	0.089	131	2.17
	0.85	8.7	0.082	29.8	2.27
	0.70	8.2	0.074	10.2	2.29
	0.55	6.2	0.065	8.42	2.40
	0.40	4.7	0.054	1.71	2.51
	0.25	5.7	0.042	0.500	2.40
	0.25	6.1	0.042	3.40	2.37
350	0.030	2.8	0.012	0.121	2.63

Ni-NiO, UO_2^{cryst}

T(°C)	NaCl(m)	HCl,(m) 10^{-3}	aCl^- (T)	U(m), 10^{-7}	pH (25 °C)
250	1.50	68	0.41	14.1	1.40
	1.40	68	0.39	20.4	1.40
	1.30	66	0.38	11.2	1.41
	1.20	67	0.36	17.0	1.40
	1.00	66	0.32	5.62	1.41
	0.95	64	0.31	14.1	1.41
	0.90	65	0.30	6.17	1.40
	0.85	63	0.29	6.03	1.41
	0.80	65	0.28	6.03	1.40
	0.75	63	0.26	7.59	1.41
	0.70	61	0.25	4.17	1.42
	0.65	49	0.24	1.41	1.51
	0.60	49	0.23	2.04	1.51
	0.55	47	0.21	0.708	1.52
250	0.50	36	0.20	0.275	1.64
300	1.50	45.5	0.26	1.21	1.57
	1.40	43.2	0.25	0.892	1.59
	1.30	44.8	0.24	1.022	1.58
	1.20	43.2	0.23	0.711	1.59
	1.00	42.9	0.21	0.715	1.59
	0.95	42.1	0.20	0.671	1.59
	0.90	42.4	0.19	0.421	1.59
	0.85	42.2	0.19	0.526	1.59
	0.80	42.1	0.18	0.458	1.59
	0.75	42.3	0.17	0.489	1.58
	0.70	42	0.17	0.420	1.58
	0.60	41.8	0.15	0.370	1.58
	0.50	41.5	0.14	0.270	1.57
	0.40	41.1	0.12	0.349	1.57
300	0.30	41.4	0.10	0.213	1.55
350	1.50	34	0.11	0.467	1.70
	1.40	38	0.11	0.237	1.65
	1.30	34	0.10	0.362	1.70
	1.20	36	0.098	0.306	1.67
	1.00	38	0.089	0.378	1.64
	0.95	37	0.087	0.308	1.65
	0.90	39	0.085	0.585	1.62
	0.85	35	0.082	0.240	1.67
	0.80	37	0.080	0.239	1.64
	0.75	39	0.077	0.429	1.62
	0.70	35	0.074	0.166	1.66
	0.65	35	0.071	0.279	1.66
	0.60	33	0.068	0.130	1.68
	0.55	31	0.065	0.143	1.70
350	0.50	35	0.062	0.299	1.65

Co-CoO, UO_2^{cryst}

T(°C)	NaCl(m)	HCl(m), 10^{-3}	aCl^- (T)	U(m), 10^{-7}	pH (25 °C)
250	0.50	40	0.32	0.509	1.53
250	0.75	49	0.26	7.20	1.45
250	1.00	54	0.20	14.2	1.41
350	0.50	27	0.089	0.218	1.70
350	0.75	34	0.077	0.470	1.60
350	1.00	34	0.062	0.705	1.61

Supplementary Table 2. The logarithm of the oxygen fugacity of the buffers employed in the experiments at 250-350 °C. The Co-CoO buffer was used at 250 °C and 350 °C, the MoO_2-MoO_3 buffer at 300 and 350 °C, and the Ni-NiO buffer at all three temperatures.

T(°C)	250	300	350
MoO_2-MoO_3	-26.5	-23.5	-20.7
Ni-NiO	-40.7	-36.3	-32.3
Co-CoO	-42.0	-37.5	-33.6
